# Nutraceuticals and pharmacological to balance the transitional microbiome to extend immunity during COVID-19 and other viral infections

**DOI:** 10.1186/s12967-024-05587-9

**Published:** 2024-09-18

**Authors:** Anju Kaushal

**Affiliations:** Auckland, New Zealand

**Keywords:** COVID-19, Gut microbiota, Dietary fibres, Vaccines, Antibiotics, Vitamins

## Abstract

**Scope:**

The underlying medical conditions and gut dysbiosis is known to influence COVID-19 severity in high-risk patients. The current review proposed the optimal usage of nutraceuticals & pharmacological interventions can help regulate the protective immune response and balance the regulatory functionality of gut microbiota.

**Summary:**

Many studies have revealed that the probiotic interventions viz., *Lactobacillus rhamnosus*, *L. plantarum* & other bacterial spp. reduce IFNγ & TNF-α and increase IL-4 & IL-10 secretions to control the immunostimulatory effects in upper respiratory tract infection. Dietary fibres utilized by beneficial microbiota and microbial metabolites can control the NF-kB regulation. Vitamin C halts the propagation of pathogens and vitamin D and A modulate the GM. Selenium and Flavonoids also control the redox regulations. Interferon therapy can antagonize the viral replications, while corticosteroids may reduce the death rates. BCG vaccine reprograms the monocytes to build trained immunity. *Bifidobacterium* and related microbes were found to increase the vaccine efficacy. Vaccines against COVID-19 and season flu also boost the immunity profile for robust protection. Over all, the collective effects of these therapeutics could help increase the opportunities for faster recovery from infectious diseases.

**Conclusion:**

The nutraceutical supplements and pharmacological medicines mediate the modulatory functionalities among beneficial microbes of gut, which in turn eliminate pathogens, harmonize the activity of immune cells to secrete essential regulatory molecular receptors and adaptor proteins establishing the homeostasis in the body organs through essential microbiome. Therefore, the implementation of this methodology could control the severity events during clinical sickness and reduce the mortalities.

## Introduction

The pathophysiological conditions and hyperinflammatory immune responses are the main concerning areas in terms of exploring the COVID-19 severity. The pre-existing comorbid conditions such as, diabetes mellitus, rheumatoid arthritis, angioimmunoblastic lymphadenopathy with dysproteinemia, cardiovascular diseases, neurological disorders, other inflammatory disorders and underlying conditions due to misuse of antibiotics etc., are known to increase the susceptibility to dysbiosis, resulting in prolonged hospitalization in COVID-19 patients escalating mortality rates [1, 2]. Microbiome research has attained great attention in the past decade because of its beneficial attributes. Bacterial species like, *Coprobacillus*, *Clostridium*, *Firmicutes*, *Bacteroides*, *Proteobacteria* and *Actinobacteria* influence the SARS-CoV-2 infection subsequently increase the disease severity. The altered gut microbiota due to COVID-19 may flare up the early induced ‘Cytokine Storm’ elevating the levels of IL-1β, IL-6, tumour necrosis factor-α (TNF-α), monocyte chemoattractant protein-1 (MCP-1), and macrophage inflammatory protein-1α (MIP-1α) and Chemokine C–C motif ligand 3 (CCL3) [1, 3, 4]. Additionally, the psychological wellbeing of patients can also be impacted via. crosstalk between gut and brain during illness and dysbiosis [5].

Interferons also provide immune modulatory response to suppress the early viral infection and regulate the α-defensin [6, 7]. Similarly, corticosteroids (glucocorticoids) suppress the initial infection and have found to control the 62% death rate, but eventually reduced to 28% in patients on ventilators [8, 9]. Effectiveness of vaccines are also described in literature to attenuate the gut microbiome restoring the essential commensals that activate NOD2 sensors and IgA production contribute to restore the intestinal barrier function [2, 10].

Post acute COVID-19 syndrome is described as the chronic form of COVID-19 could last for 6 months or years. The mood disorders and autoimmune dysfunctions manifested as a consequence of infectious disease, genetic, environmental and socioeconomic problems, are the predisposing factors towards developing chronic fatigue syndrome. PACS is likely to be contexed with chronic distress and brain fog with cognitive impairment. The post infection fatigue syndrome has also been documented in case of influenza, dengue, Epstein-Barr virus, enteroviruses, human parvovirus and protozoan infections [5, 11]. Lack of important nutrients e.g., vitamin C, B, D, sodium and magnesium, zinc, folic acid etc. may worsen the illness [5].

The viruses and bacteriophages distribute the essential regulatory genes required for vital activities and genetic make-up of microflora. Antibiotics associated dysbiosis occurred can be repaired with the help of appropriate probiotics eventually confer the better performance of microbiome, hence, mitigating the risks of poor disease prognosis. WHO had already provided the plans for food and nutrition to people who were supposed to go through the self-quarantine during COVID-19 outbreak in order to strengthen the immunity, irrespective of their acquired disease status. Following the diet regimens including dietary fibres, omega-3-polyunsaturated fatty acids (ω-PUFA), polyphenols, vitamin A, C, D etc. along with probiotics, could help offset the ‘cytokine storm’ formation. The bioactive compounds, however, must be optimally evaluated and employed for the management of viral diseases and associated chronic fatigue syndrome. The nutraceutical supplements, pharmacological therapeutics and their interactions with microbiota is supposed to unveil the positive immunological activities could impact the disease outcomes [5, 12–17].

This review emphasizes the values of optimal use of nutraceuticals (active compounds) and pharmacological sources to moderate the immune response by controlling the hyperactivated macrophages/monocytes to halt the excessive production of cytokines and chemokines. Consequently, these events help release the molecular adaptors, which program the chromatin functionality via histones at transcriptional level to impact the post translational modifications during cell differentiation. Undoubtedly, this approach would be a step forward for the earlier & faster recovery of both asymptomatic and symptomatic patients.

## Effects of nutraceuticals

### Probiotics

Food and Agriculture Organization (FAO) and World Health Organization (WHO) have defined ‘Probiotics’ as microbes confer the health benefits to the host [18–20]. Probiotics (*Lactobacillus*, *Bifidobacterium*, and *Saccharomyces* spp*.*) and prebiotics (non-digestible food ingredients) attenuate the microbiota and increase GM’s survival [20, 21]. By preserving the gut microflora through bioactive food supplements increase the capability of immune cells to control cytokines’ release, TNF-α and activate the reactive oxygen species (ROS) to establish optimal anti-viral state [21]. The probiotic supplements have been experimented on ARDS and pneumonia in COVID-19 patients on ventilation has shown significant improvement [1]. *L. rhamnosus* GG is safer and efficacious in preventing viral associated pneumonia (VAP) in high-risk ICU population [22, 23]. However, there is still lack of sufficient peer reviewed information on the probiotic supplements in controlling the ventilation associated pneumonia [24].

**Fig. 1:**
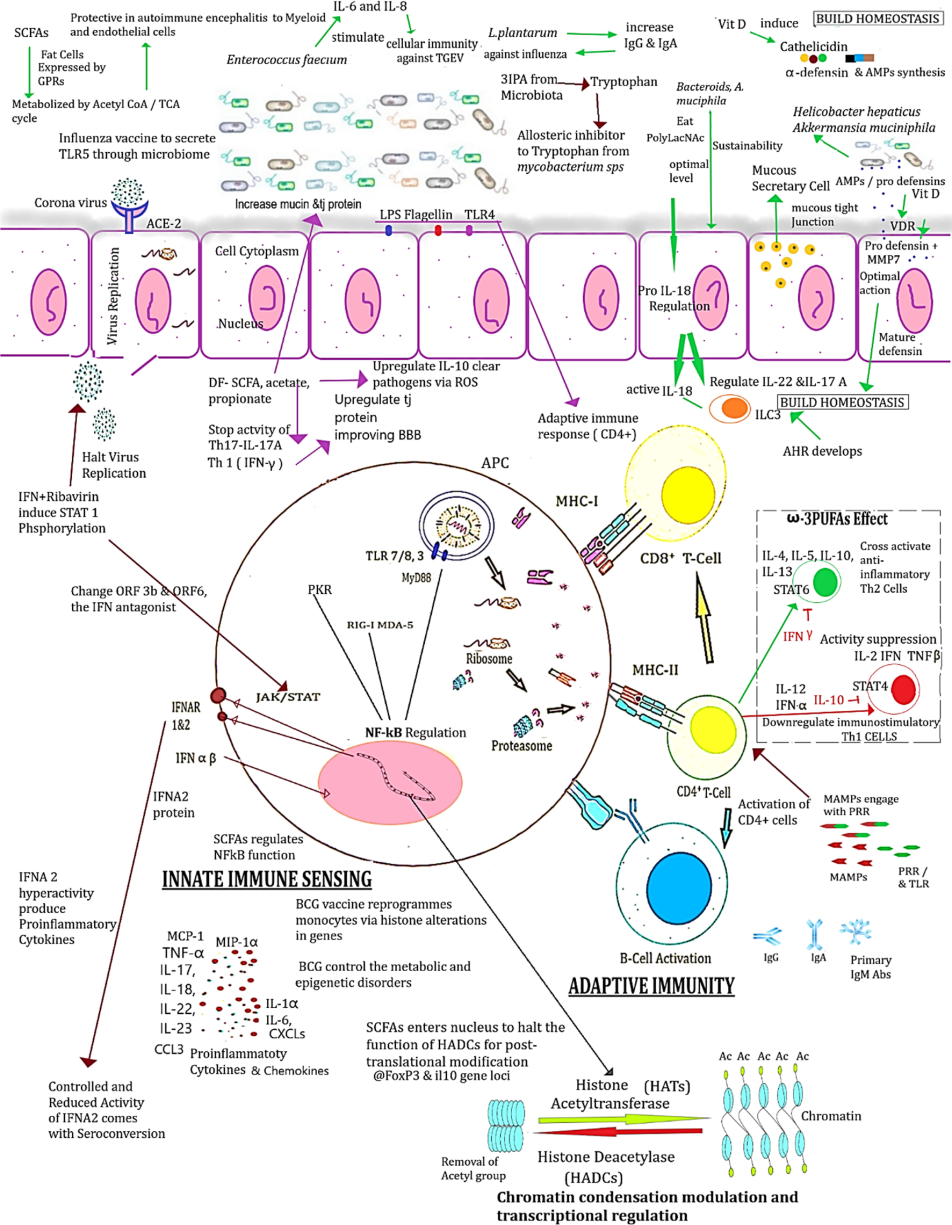
Illustration of the molecular mechanisms developed during administration of pharmaceutical interventions and nutraceutical supplements [2, 5, 16, 21, 25, 27, 28, 32, 33, 36–39, 66, 67, 69, 73, 74, 78, 83, 84, 95]. Bacteroides via glycolysis to produce SCFAs & other acetates are utilized by other commensals through TCA giving innate protection to myeloid cells. SCFAs regulate IL-10 and halt IL-17A, & via Th1 activating ROS to clear pathogens. Influenza vaccine regulates TLR5. Vit D via VDR activates the cathelicidin, α-defensin and AMP synthesis against pathogens. Microbiota secreted 3-IPA acts as an allosteric inhibitor to tryptophan of *Mycobacterium tuberculosis* & *non-mycobacterial* spp. ω-3 PUFAs indirectly activate Th2 cells to regulate IL-4, IL-5, IL-10, IL-13 via STAT-6 pathway, by repressing Th1. IFN+ Ribavirin regulates IFN & change IFN agonist e.g., ORF 3b and ORF 6. MAMPs and PRRs regulate the CD4+ cells. Deactivation of IFNA2 is correlated with seroconversion. BCG vaccine reprograms the monocytes through histone alterations inhibiting HADCs activity to control the metabolic and epigenetic disorders building trained immunity. *Bacteroides* utilized mucin for longer survival and regulate pro-IL-18 & ILC3. *Enterococcus faecium* to stimulate IL-6 & IL-8 to fight against TGEV

The cellular immunity is increased using *Enterococcus faecium* NCIMB 10415 that provokes IL-6 and IL-8 secretion to fight against the TGEV infection in young piglets. European food safety authority has developed a guidance to use *E. faecium* in dose dependent manner in animals [25]. Certain *Bacteroides* spp. and *Akkermansia muciniphila* are known to utilize mucin polyLacNAc via *O*-glycanases & glycoside hydrolases for their continual survival. However, the excessive usage of mucin could allow pathogens to grow faster to raise the alarm for inflammatory bowel disease (IBD) and even colorectal cancer [26]. Table 1 and Fig. 1 represent the comprehensive details on the characteristics and molecular mechanisms of microbiota. The bioactive food supplements, prebiotics, and synbiotics can be utilized as adjunctive therapeutics, attributed to control the secondary infections too [27]. The legitimate safety and efficacy of probiotics for the regulatory point of view is yet to be investigated against infectious diseases.

**Table 1:** Immunomodulatory and pathological implications to fight against diseases using favourable microbes and nutraceuticals (dietary fibres, vitamins, polyunsaturated fatty acids and flavonoids)

Relevant features	Modulatory/pathological factors	References
Bioactive supplements (prebiotics, probiotics & synbiotics)		
Bioactive food supplements with probiotics increase the survival of commensals Restore microflora diversity	TLR modulation, balance the immune response to confer viral clearance, boost vitamin's absorption **↓**Allergic reactions & pulmonary symptoms **↑** Lung’s protection, TLR modulation, viral clearance & vit. absorption	[2, 5, 18, 19, 22–25, 27, 86–94]
*Lactobacillus*, *Bifidobacteria,* and *Saccharomyces* spp. are the mainly used as probiotics	Can control the ventilation associated pneumonia and the cytokine storm with ARDS in COVID-19 patients	
BP-38 catabolic activator from *Paenibacillus *spp.	Suppress ACE-2 function (Mice model)	
*E. faecium* can treat intestinal cells	Stimulate IL-6 and IL-8 to raise cellular immunity against TGEV infection	
Capsular polysaccharide A-*Bacteroides fragilis*	Induce IFNβ in-vitro and DCs in lamina propria of colon to resist against viral infection	
*Parabacteroides*, *Bacteroides*, and *Lachnospiraceae* families	Help synthesize SCFAs to modulate the immune cells, balance the innate response, and maintain the integrity of mucosa	
*Lactobacilli and Bifidobacterial *spp.	Used in antibiotic associated diarrhoea	
*Blautia wexlerae* and *Bifidobacterium longum* with other bacterial spp. were negatively associated with post-acute covid syndrome PACS at 6 months	Microbiota modulation may assist in early and safe recovery minimizing the risk of developing PACS	
Probiotics formulated (450 billion bacteria) used specific strains of *Bifidobacterium breve* DSM24732, *Streptococcus thermophilus* DSM 24731, *Bifidobacterium infantis* DSM 24737, *Bifidobacterium longum* DSM 24736, *Lactobacillus acidophilus* DSM 24735, *Lactobacillus paracasei* DSM 24733, *Lactobacillus plantarum* DSM 24730, *Lactobacillus delbrueckii subsp. * * bulgaricus* DSM 24734	Patients with familial Mediterranean fever FMF (inter-critical period) were observed with improved symptoms	
*L. plantarum* DR7	↓ IFNγ & TNF-α concentration, and ↑ IL-4 and IL-10 in young adults with upper respiratory infection	
*Lactobacillus casei* DN-114001 used in clinical controlled trial	↓ episodes of upper respiratory tract infections within 3 months	
*Lactobacillus rhamnosus* GG	↓ hospitalization, control the imbalance in microbiota richness induced by antibiotics and ↑ microbiota diversity	
Dietary fibres (DFs)		
DFs help maintain the gut microbiome composition	hsCRP, IL-6, TNF-α induction is reduced, control the inflammation	[2, 5, 12, 13, 28–31, 36]
Phenolic compounds of DFs influence the diversified growth of gut microbiota with *Lactobacillus, Bifidobacterium, Akkermansia* and *Faecalobacterium*	Inhibit *Helicobacter pylori* and *Staphylococcus* spp.	
SCFAs synthesized from *Bifidobacterium *spp. and *Lactobacillus* spp.	↓ growth of pathogen like C*lostridium* spp.	
SCFA related GPCRs activation, increase the signaling of anti-inflammatory cascade	↑ IL-12 inhibition and IL-10 upregulation	
Exopolysaccharides- homopolymers-cellulose, levan, curdlan, dextran	↓ TNF-α & IL-1 and NF-kB hyper-production	
Heteropolymers- xanthan, gellan, galactan, kefiran are produced by *Lactococcus lactis, Lactobacillus *spp.,* Streptococcus thermophilus, Weissella confusa, Leuconostoc* spp., and* Pediococcus* spp.	Modulate CD8+ and Tc17 cells and↑ anti-inflammatory properties	
Enzymes (oxidoreductase, transferases, hydrolases, lyases, isomerases, ligases) secretion from *Bacillus subtilis, Bacillus licheniformis, Aspergillus niger,* * Aspergillus oryzea and Lactobacillus lactis*	Immunomodulatory, antitumor, antimutagenic, anti-oxidant, anti-inflammatory, anti-hypertensive, antibacterial and antiviral, cholesterol lowering, anti-IBD properties	
Cell wall components (Peptidoglycan-Teichoic acid and Lipoteichoic acid) especially from gram+ ve bacteria e.g., *Lactobacillus* spp.	Promote physiological, biochemical and regulatory properties	
Cell Free supernatant and bacterial lysate from *Lactobacillus* spp. and *Bifidobacteriums pp.*	Immunostimulatory and safe to human health except biogenic amino acids and d-lactis acid	
Vitamins		
Vitamin C (l-ascorbic acid-2-glucoside)	Neutralise the oxidative stress, apoptotic response, decrease the cellular viability in *H. pylori* Also inhibit HSV, polio, and influenza	[5, 40, 42–46]
Vitamin D&E	*Bifidobacterium, Lactobacillus, Akkermansia, Roseburia* to get modulated. Could reduce Firmicutes/Bacteroides (F/B) ratio, if used inappropriately	
Vitamin E with Selenium and retinoic acid	Ameliorate mucosal inflammation, ↑ *Bacteroidetes* and ↓ *Firmicutes* (mice model). Inhibit Norovirus replication, but ↑ *Lactobacillus* spp. abundance	
Vitamin A exhibits adjuvant properties	Retinoic acid vit A can cause the hepatotoxicity, if used inappropriately	
Vitamin D	Immunomodulatory/and anti-inflammatory effects on immune cells ↓ hospitalization potentially while maintaining the adequate level (> 20 to < 50 ug/ml). Administered at higher doses can reduce ICU admissions	
Vitamin K deficiency	Poor prognosis of COVID-19. Moderate level of Vit K suppresses the co-infections with COVID-19 with increased use of antibiotics	
Essential trace minerals—selenium & zinc		
Selenium increases the beneficial microbes such as, *Akkermansia, Lactobacillus,* and *Faecalbacterium* and depletes the deleterious microbes like *Dorea* and *Mucispirillum*	Gut microbiota to secrete SCFAs to control the inflammatory bowel disease (IBD) and colitis, by decreasing the GM impairment Regulates the redox state	[29, 35, 47–50]
Zn is essential for the normal functioning of the immune system	Combines with ionophore pyrithione help inhibit the SARSCoV-1, polio and influenza replication Inhibit RNA synthesis by inhibiting the activity of enzyme RdRp	
Zn & Selenium supplementation at non-toxic level	Binds S100 protein fecal-calprotectin exert anti-microbial effects against *C. difficile* Strengthen the immune system	
Polyunsaturated fatty acids		
ω-3-PUFA influence the immune cells activation	Metabolize into prostaglandins, leukotrienes, thromboxanes, maresins, protectins and resolvins used by immune cells SCFA & butyrate producing microbial growth is increased with *Bifidobacterium, Lachnospira, Roseburia*, and *Lactobacillus* ↓ risk of hospitalization 12–20% in COVID-19 patients Downregulate the IL-2 driven CD4+ and CD8+ T-cell activation Indirect suppression of Th1 cells, increase the cross regulatory function of Th2 producing anti-inflammatory effects	[14–16, 51, 77]
Flavonoids		
Flavonoids help treating the viral infections	↑ Treg and T17 cells to inhibit the pathogens	[17, 52]
Increase *Bifidobacterium *spp. and *Lactobacillus* spp.	Suppress the TLR4, 1kB phosphorylation to decrease the nuclear translocation of NF-kB	
Halt the growth of *Clostridium *spp.,* Helicobacter pylori*, *Escherichia coli,* and *Salmonella typhimurium*	Antioxidant properties of flavonoids protect against the cardiovascular, metabolic and neurodegenerative disorders

### Dietary fibres (DFs)/prebiotics

Dietary fibres (DFs) are the plant derived foods utilized by microbes to yield the beneficial byproducts like SCFAs. These metabolites activate the anti-inflammatory cascade through G-proteins coupled receptors (GPRs) that repress IL-2 and upregulate IL-10 in monocytes. SCFAs also halt TNF-α, IL-1, but regulate nuclear factor kappa-light-chain-enhancer of activated B cells (NF-kB) [28]. The consumption of DFs (5 g/day) reduces the level of C-reactive protein (hsCRP), IL-6, TNF-α etc. Therefore, SCFAs promote migration of immune cells near inflammatory sites to clear the pathogens via. ROS to lower the systemic and gut inflammation (Table 1) [28–30]. The enrichment of diversified beneficial bacteria such as, *Bifidobacterium *spp. and *Lactobacillus *spp. inhibit the growth of detrimental pathogens like *Clostridium *spp. The phenolic compounds rich DFs promote the propagation of *Lactobacillus*, *Bifidobacterium*, *Akkermansia*, and *Faecalibacterium*, but suppress the growth of *Helicobacter Pylori* and *Staphylococcus *spp. [31].

### Postbiotics

The postbiotics are the metabolic byproducts of commensals vitally used for many physiological and metabolic functions to maintain the homeostasis effectively [13]. The SCFAs, mainly constitute acetate, propionate and butyrate, are recognized by GPRs-GPR 41 & 43. The GPR 41 and GPR 109 in adipose tissues metabolize into acetyl CoA regulating TCA cycle and cell function [32]. The colonic low pH, and serum lipid profile are also restored by SCFAs. Overall, effective use of SCFAs strengthen the immune system fighting with pulmonary diseases, hence, reducing the mortalities [28, 33, 34]. SCFAs also increase the TjP integrity against pathogens invasion [35].

SCFAs inhibit the activity of histone deacetylase (HDAC) to control the process of deacetylation and histone crotonylation involving in the post-translational modification facilitating the gene changes, hence, regulate the chromatin architecture critical for epigenetic modification during cell cycle and differentiation [36]. The indole and IPA also modulate the cells to produce antibodies (Fig. 1). Exopolysaccharides-homopolymers (cellulose, levan, curdlan, and dextran) and heteropolymers (xanthan, galactan, kefiran) are the biproducts of *Lactococcus *spp.,* Streptococcus *spp.,* Leuconostoc *spp., that downregulate the IL-1 and TNF-α and upregulate CD8+ and T17 cells (Table 1). Various enzymes like, oxidoreductase, transferase, hydrolases, lyases, isomerases being secreted from *Bacillus subtilis, Bacillus licheniformis*, *Aspergillus niger*, and *Aspergillus oryzae*. *Lactobacillus lactis* provides every possible aspect of maintaining the homeostasis through anti-inflammatory, antitumor, anti-cholesterol and antioxidant response (Table 1). Physiological and metabolic pathways are also regulated using cell wall components and cell free components of *Lactobacillus *spp. [12, 13].

The mucin degrading bacteria target the polyLacNAc structures with in oligosaccharide side chains in both humans and animals. These *O*-glycanase enzymes are the glycoside hydrolase16 (GH16) family indicating their role in mucin break down. This administers the advanced knowledge of mechanisms of mucin breakdown by normal microbiota, could provide a research tool to explore *O*-glycan for intestinal diseases. *Bacteroides *spp. and *Akkermansia muciniphila* utilize mucins for their long -term survival. However, the excessive usage of mucin could allow pathogens to grow faster raise the alarm for inflammatory bowel disease (IBD) and even colorectal cancer [26].

The bioactive food supplements, prebiotics, and synbiotics can be utilized as adjunctive therapeutics, as these modulate DCs and regulate IFNs. Besides, repairing the altered microbiota to boost immunity against SARSCoV-2 infection, it also assists in controlling the secondary viral and bacterial infections [27, 37]. Pathological and modulatory events using probiotics and pre and post biotics are explained in Tables 1 and 2.

**Table 2:** Pharmacological interventions to influence the immune modulatory/or pathological events via. microbiota

Relevant characteristics	Immune modulatory/pathological events through microbiota	References
Interferons		
IFN-α and IFN-β harness anti-SARS and anti-MERS properties IFN-β with ribavirin IFN effects on Calu3 2B4 cells Interferon tau	Immune-modulatory effects on innate immune system Protect susceptible and severely ill patients Could suppress the virus replication & transmission ↓ SARSCoV-2 replication. Induce the STAT-1 phosphoryaltion, ORF3b and ORF6 changes couldn’t antagonize IFN Changes GM and IL-17, protect against influenza (Mice) ↓ *Firmicutes* and ↑*Bacteroides* in jejunum and ileum	[7, 53, 54, 56, 57, 79]
Corticosteroids		
Corticosteroids (DEX) are used in asthma, allergies and autoimmune disorders GM gets altered using DEX, retaining *Bifidobacterium* and *Lactobacillus* (For anti-inflammatory effects- mice model)	Influence physiological functions, reduce massive lung damage, activate transcription factor- TF to regulate NF-kB and AP-1 to decrease proinflammatory cytokine production Synthetic glucocorticoids methylprednisolone administration in ARDS COVID-19 patients could decrease the risk of death by 62% Long-term use—cause lipid accumulation & upregulation of GC receptor to cause circadian rhythm disorder	[59–63, 80]
Antibiotics		
Antibiotics treatment to combat the secondary infection during COVID-19 could kill gram −ve/+ve gut microflora Broad spectrum antibiotic perturbation	Alter gut microbiota and cause gut dysbiosis, dysregulation of DCs activation ↑ tissue pathology by CX3CR1 + in mononuclear cells ↓ *Bacteroidetes*, Increase the circulatory inflammatory signatures ↓ expression of IFN-γI, MHC-I, CD40 during the early viral infection and ↓ modulated GM ↑ secretion of IFNγ, IL-6, CCL-2 and ↓ Treg cells, cause permanent dysbiosis	[64, 81, 82, 90]
Vaccines		
BCG vaccine (live-attenuated) produces heterologous immune response against Tuberculosis and other infections *Bifidobacterium adolescentis* immunomodulates the response *Bifidobacterium adolescentis, Butyricimonas virosa, Adlercreutzia equolifaciens* and *Asaccharobacter celatus* *Ruminococcus torques, Eubacterium ventriosum* and *Streptococcus salvarius* *Prevotella copri* and *Megamonas* species *Prevotella copri* *Megamonas funiformis*, *Megamonas hypermegale*	Modulates the immune response against the metabolic and epigenetic disorders Flagellin and peptidoglycan are adjuvant in nature TLR-4 activated by LPS to ↑ adaptive response TLR5 are sourced from microflora activate the response against influenza and polio vaccine ↑ response against CoronaVac vaccine, but single dose with BNT162b2 generated a ↓ level of neutralizing titre *Bifidobacteria* potentiates the carbohydrate driven immunogenic effects for ↑ Ab production Beneficial effects on NAb immune response generated using CoronaVac vaccine, also affect the body weight and BMI in CoronaVac vaccine ↑ response to vaccine ↓ adverse effect and ↑ anti-inflammatory Linked with farnesoid X receptor signaling via modulating bile acid metabolism Ferment glucose into acetate and propionate to maintain homeostasis, regulate T17 helper cells	[66–72]

### Vitamins

Vitamins have adjuvant properties and restore health beneficial bacteria e.g., *Bifidobacterium, Lactobacillus, Akkermansia*, and *Roseburia*. The micronutrients execute anti-oxidant function to attenuate immunostimulatory inflammation. Vitamins/antioxidants may alter the circulatory ROS and levels of antioxidant enzymes such as, superoxide dismutase SOD, catalases, peroxiredoxins-PRXs, glutathione peroxidases-GPXs etc., which follow the ascorbate glutathione pathway. Vit. C, D and E can reduce the Firmicutes and Bacteroides (F/B) ratio. Vitamin K deficiency could lead to the poor prognosis of COVID-19. Vit. D via VDR receptors, induces the antimicrobial cathelicidin in macrophages and synthesis of α-defensin & antimicrobial peptides (AMPs), and reduce the production of inflammatory cytokines (Table 1 and Fig. 1) [5, 38]. The interventional studies have shown that vit. D alters the microbiota composition by increasing the beneficial microbes viz., *Ruminococcus*, *Akkermansia*, *Faecalibacterium*, *Lactococcus*, and *Coprococcus*, while decreasing Firmicutes. Hence, maintaining the appropriate level of vitamin D helps balancing the microbiota [39]. Vitamin D is administered in higher dose significantly reduce the risk of ICU admissions in COVID-19 cases [40]. The hospitalized patients (84%), with COVID-19 were reported to deficient in vit. D [25 (OH) D], were associated with higher D-dimer level, could be considered as a factor for disease prognosis. Maintaining the adequate level of vit. D (> 20 to < 50 ug/ml) could reduce the direct hospitalizations, non-invasive ventilation support and ICU admissions [41, 42].

Vitamin C could provide the non-specific protection against the respiratory infections with *Mycobacterium tuberculosis, Pseudomonas aeruginosa*, heamolytic *Streptococci, Staphylococcus aureus* MRSA, *E. faecalis*,* E. coli* O 157: H7, *Klebsiella pneumoniae* and *Proteus mirabilis*. Supplementation with vitamin C, D and vitamin E in the nutritional sources facilitate the growth of *Bifidobacterium, Lactobacillus,* and *Roseburia* to control the lower ratio of *Firmicutes/Bacteroides*, consequently the beneficial gut microbes’ abundance is enhanced [21, 28]. l-ascorbic acid-2-glucoside, a form of vitamin C could neutralize/or counteract with oxidative stress, apoptotic responses, and decreased cellular viability caused by *Helicobacter pylori*. It also holds the anti-Campylobacteriosis and anti-Salmonellosis properties. An oxidized form of vit. C dehydroascorbic acid could inhibit the replication of herpes simplex virus type 1, polio virus type 1 and influenza virus type A and so as to reduce the parasite count too in case of Plasmodium and Trypanosoma. Vitamin D is associated with inhibition of Hsp90-mediated proteostasis which governs morphogenesis, by repressing the cyclic AMP-protein kinase A in *Candida albicans* [30, 43].

**Fig. 2:**
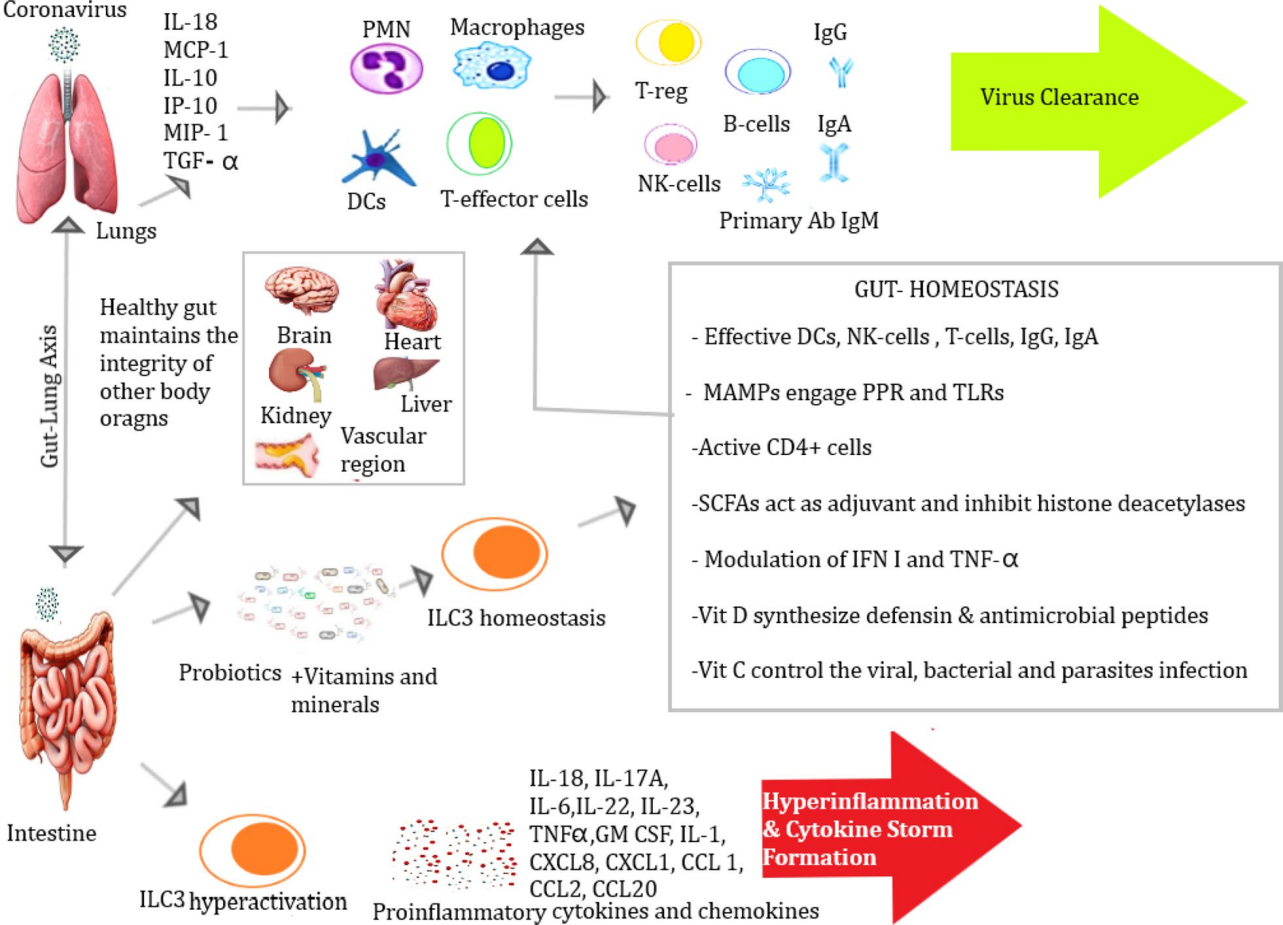
Properties of Nutraceuticals and Pharmacological interventions to regulate the protective immune activities in alveolar and gut cell linings. A probable modulatory effect of probiotics with supplements to build ILC 3 homeostasis in gut and the functional integrity of the other body organs [2, 20, 29–32, 36]

Vitamins also modulate the health beneficial bacteria e.g., *Bifidobacterium, Lactobacillus*, *Akkermansia*, and *Roseburia*. Vitamin D and E can control the *Firmicutes* and *Bacteroides *(F/B) ratio (Fig. 2, Table 1). Vitamin E with selenium and retinoic acid induces the restoration of gut microflora to increase *Bacteroidetes* and decrease *Firmicutes* (mice model), thus ameliorating the mucosal inflammations [44]. Adjuvant nature of vit. A inhibits norovirus replication and increases *Lactobacilli* abundance [45]. Bile acids and microbiota contribute to optimize the level of vitamin K [46]. But vitamin K deficiency could lead to the poor prognosis of COVID-19.

### Selenium, zinc and ω-3 PUFAs

Selenium (Se) is an essential trace mineral required for selenocysteine synthesis, catalysed by glutathione peroxidase (GPxs), thus, having a significant role in redox state regulation. It depletes the deleterious microbes like *Dorea* and *Mucispirillum*, but increase the beneficial microbes like *Akkermansia*, *Lactobacillus*, and *Faecalibacterium* [29, 47, 48].

Zinc (Zn) and Selenium help strengthen the immune system. Zn controls the level of inflammatory mediators, activity of O2 and N2, which destroy the host tissues. Ionophore pyrithione blocks SARSCoV-1, polio and influenza replication [49]. Zn-pyrithione could halt the growth of deleterious microbes interfering with copper flux and inhibit the ionophore loading in nucleus. Zn also showed a calprotectin-mediated antimicrobial effects in *C. difficile* [50].

Omega-3 polyunsaturated fatty acids (ω-3 PUFAs) and Omega-6 fatty acids intake could reduce 12–21% risk of COVID-19, hence, reduce hospitalization and ventilation. However, in critical cases the higher level of fatty acids has resulted in the formation of ‘cytokine storm’ in lungs increasing the mortality. Therefore, it is imperative to investigate the risk of dose level with Omega 3 fatty acids [15]. ω-3 PUFAs augment the production of SCFAs through *Bifidobacterium*, *Lachnospira*, *Roseburia*, and* Lactobacillus* [14, 51].

### Flavonoids

Flavonoid compounds have antioxidant properties to provide protective effects on broad range of disorders viz., cardiovascular, metabolic and neurodegenerative. Antioxidant compounds increase the growth of symbiotic microbes *Bifidobacterium *spp. and *Lactobacillus *spp., while hindering the growth of *Clostridium *spp., *Helicobacter pylori*, *Escherichia coli*, and *Salmonella typhimurium* [17, 52]. Flavonoids and microbiome interactions could be helpful in the treatment of viral infections. Flavonoids increase Treg and T17 cells and inhibit the pathogens to propagate. They also suppress the TLR4, reduce the 1kB phosphorylation to decrease the nuclear translocation of NF-kB [28, 36]. A microbial phenolic acid, Desaminotyrosine (DAT), is secreted by *Clostridium orbiscindens* helping rescued the antibiotic treated influenza-infected mice, via 1 IFN signaling increment stopping the lung pathology [53].

## Pharmacological interventions

### Interferons

Type I IFNs (IFN-α and IFN-β) exert immunomodulatory effects and antagonize the replication of SARSCoV-1 and MERS-CoV [2, 54]. The synergistic effect of IFN-β with ribavirin suppresses the early phase viral replications, therefore, reducing the risk of transmission [55]. The IFN tau supplement increases *Bacteroides* & decreases *Firmicutes*, expressing IL-17 to protect mice [6, 56]. Similar results were obtained in multiple sclerosis patients receiving IFNβ-1b, would warrant more developmental studies for further justifications [7]. However, IFNβ-1a has given the potential safety effects to treat COVID-19 patients (NCT04343768) [57]. IFN-γ administration induces signal transducer and activation of transcription 1/&3 (STAT1/& STAT 3) to upregulate Reg3 and α-defensin to maintain homeostasis against alcohol induced intestinal disruption and elevated endotoxin level and hepatic inflammation (mice model) [58].

### Corticosteroids

The corticosteroids influence NF-kB and activator protein (AP-1) to modulate their effects to activate the transcription of anti-inflammatory factors and reduce the proinflammatory cytokines production, therefore, offset the ‘cytokine storm’ formation [59]. Corticosteroids based therapies were used in SARS outbreak in 2003, and are being used in COVID-19 patients too. A synthetic glucocorticoid methylprednisolone administration in ARDS patients could decrease the risk of death by 62%, but not in all cases [8]. In contrary, the corticosteroids also reported to increase the clinical symptoms, inflammation and computed tomography (CT) abnormalities, could remain non-effective during prolonged ventilation and prompt the secondary infections [60]. Dexamethasone (DEX), 9-fluoro-glucocorticoid, acquires anti-inflammatory and immunosuppressive attributes found to reduce death rates 30% in patients on ventilation and is being widely used to treat asthma, allergies and autoimmune disorders (Table 2) [61]. Glucocorticoids influence the gut physiological functions and help retain the growth of *Bifidobacterium* and *Lactobacillus* bacteria in mice [62]. But higher dose level of corticosterone upregulates the GC receptor expression in peripheral tissues may cause physiological alterations and circadian rhythm disorder [63]. The effectiveness of corticosteroids on COVID-19 patients remains conclusive. A meta-analysis of large multinational recovery trials recruiting 20,197 patients (44 studies) using corticosteroids, have shown 28% reduction in mortality rates. However, in another trial with 4451 patients revealed the increasing mortality risks by twofolds [9].

**Table 3:** The interventional studies are being carried out using probiotics in COVID-19 individuals with expected outcome (ClinicalTrials.gov—U.S. National Library of Medicine)

Clinical trial (randomized &controlled studies)	Title of the study	Intervention (dietary supplements)	Expected outcome
NCT04390477	The intestinal Microbiota as a therapeutic target in hospitalized patients with COVID-19 infection	1 × 10^9^ CFU probiotic with maltodextrin/one capsule for 30 days	Positive effects led to produce less severe clinical evolution of the disease with -ve PCR and antigen test
NCT04458519	Efficacy of intranasal Probiotic Treatment to reduce severity of symptoms in COVID-19 infection	Nasal irrigation with Probiorinse-2.4 billion CFU of *Lactobacillus lactis* W-136 (NPN 80085895) twice daily for 14 days	Change in the severity of COVID-19 infection ≥ 35 on visual analogue scale (VAS)
NCT04366180	Evaluation of probiotic *Lactobacillus coryniformis* K8 on COVID-19 prevention on healthcare workers	*Lactobacillus* K8(3 × 10^9^ CFU) a capsule per day during 2 months	COVID-19 preventive study on healthcare workers, including fatigue status and gastrointestinal symptoms
NCT04517422	Efficacy of *Lactobacillus plantarum* and *Pediococcus acidilactici* in adults with SARSCoV-2 & COVID-19	Probiotics *L. plantarum* (CECT 7481), *L. plantarum* (CECT 7484), *L. plantarum* (CECT 7485) and *Pediococcus acidilactici* (CECT 7483) with maltodextrin (E 1400 qs) formulated in vegetable hydroxymethylpropyl cellulose capsule	Severity progression, stay at ICU for 30 days and mortality ratio, change in viral load, IgM, IgG, fecal microbiome and serum biomakers
NCT04399252	A randomized trial of the effect of *Lactobacillus* on microbiome of household contacts exposed to COVID-19	*Lactobacillus rhamnosus* GG, 2 capsule/day for 28 days	Incidences of one or more symptoms of COVID-19 during study period, change in microbiome shannon diversity ( fecal and nasal)
NCT04420676	Synbiotic therapy of gastrointestinal symptoms during COVID-19 infection: A randomized double-blind placebo-controlled Telemedicine study (Syn CoV Study)	Omnibiotic (Probiotic mixture) AAD twice a day: *Bifidobacterium bifidum W23, B. lactis W51, Enterococcus faecium W54, Lactobacillus acidophilus W37, Lactobacillus acidophilus W55, L. paracasei W20, L. plantarum W1, L. plantarum W62, L. rhamnosus W71, and L. salivarius W24*; are mixed with maize starch, inulin, potassium chloride, hydrolysed rice protein, magnesium sulphate, fructooligosaccharide (FOS), enzymes (amylases), vanilla flavour and manganese suphate	Stool Calprotectin level measurement, duration and severity of COVID-19, stool zonulin for IBD, microbiome composition
NCT04507867	Effects of Nutritional support system to reduce complications in patients with COVID-19 and co-morbidities in stage III	Combination of Vit B1, B6 and B12, 10 mg sol./24 h for 5 days Probiotic *Saccharomyces boulardii* CNCM I-745 “Floratil” (250 mg capsule) twice a day for 6 days Nutritional support system NSS-1 in water 400 ml	Overall survival after 40 days completion, progression to mechanical ventilation, post COVID syndrome PHQ-9 test for mental disorders, organ failure assessment, cellular and biochemical tests in deceased patients
NCT04581018	An evaluation of a symbiotic formula for symptoms improvement in hospitalized patients with COVID-19 infection	Health supplements (Synbiotics daily) plus standard care for 28 days	Combined symptom score and clinical improvement, SARSCoV-2 Abs development, hospital stay, life quality, time to -ve PCR, microbiome change, mortality rate
NCT04621071	Evaluation of the efficacy of probiotics to reduce the duration and symptoms of COVID-19 (PROVID-19) study	Probiotics 2 strains (10 × 10^9^ CFU)-2 capsules (one to swallow and 2nd one mixed with maple syrup)/day for 10 days and only one closed capsule from day 11–25	Duration of symptoms to disappear and severity of COVID-19, evolution of oral fecal microbiota
NCT04666116	Change in viral load of patients with COVID-19 disease after dietary supplementation with probiotics	Probiotics	Viral load assessment during the period of admission, IL-6 analysis, mobility, microbiome analysis, for 1 year time frame
NCT04730284	Evaluation of symbiotic formula in patients with COVID-19	Health supplements tailor made synbiotics—4 g/day for 28 days	Changes in gut microbiomeβ, fecal bacteria metabolites, cytokines IL-6, IL-1β, TNF-α, CXCL-10 and trends in symptoms score
NCT04734886	Exploratory study on the effects of probiotic supplementation on SARSCoV-2 Ab response in healthy adults	*Lactobacillus reuteri* DSM 17938, 1 × 10^8^ CFU + Vit D 10 ug-2 capsule per day for 6 months	Change in SARSCoV-2 specific antibodies, IgA level, activation of innate immune molecules and adaptive immune cells.
NCT04756466	Multicentre, randomized, double blinded parallel group pilot study to evaluate the effect of consumption of *Lactobacillus* strains on the incidence of COVID-19 in elderlies	*Lactobacillus* probiotic 3 × 10^9^ CFU per day during 3 months	Incidence of SARSCoV-2 infection confirmed by PCR, IgA, IgG, fatigue status, gastrointestinal symptoms and pharmacological treatment
NCT04793997	Covid-19 Primary Care Support with Microbiome Therapy	Throat spray with 3 *Lactobacilli* strains daily for 2 weeks	Changes in severity symptoms of COVID-19 patients in 3 weeks, Ab level and change in pathogens after spray
NCT04798677	Multicomponent intervention (clinical and nutritional) resulting from the administration of influenza or COVID-19 vaccine and supplementation with ABBC-1	ABBC1 immunoessential: Yeast β glucan complex with consortium of *Saccharomyces cerevisiae* + Selenium and Zn	Alteration in acute immune response after influenza & COVID-19 vaccine ( B&T cells status) in 35 days
NCT04813718	Post COVID-19 syndrome, A-pilot study to explore the gut -lung axis	Pre and probiotic, omni-biotic pro Vi5	Microbiome composition along with other biomarkers -T & B cells, neutrophils and monocytes function test, ILs and TNF-α measurement
NCT05080244	Evaluation of efficacy of probiotics taken during the acute phase of COVID-19 to reduce the occurrence of long COVID	Probiotics: 2 strains 10 × 10^9^ CFU/capsule ( 2 capsules /day 1 closed and 1 with maple syrup for 10 days, and 1 capsule for the following 15 days)	Patients with long COVID symptoms after 90 days of COVID-19 diagnosis (COVID-19 symptoms, anxiety, functioning difficulties etc.)
NCT05043376	Study to investigate the treatment effects of Probiotic *Streptococcus salivarius* K12 in hospitalized patients (Non -ICU) with COVID-19	BLIS K12—2 tablets/day up to 14 days	Clinical improvement including inflammatory markers-recovery with live discharge
NCT04950803	A randomized-controlled Trial of an Oral Microbiome Immunity Formula in Reducing Development of Long-term Co-morbidities in Recovered COVID-19 patients	3 *Bifidobacteria* 10 billion CFU/Sachet (1 Sachet for 6 months)	A composite outcome of any comorbidity + manifestation of Long COVID, fecal microbial metabolites and inflammatory cytokines profile
NCT04941703	Investigation of choice alteration of Gut metagenome on COVID-19 severity	Magnesium citrate fluid 296 ml + probiotics 2 capsules twice daily for 6 days	Change in COVID-19 status on the ordinal outcome scale after 7 days
NCT04937556	Randomized, double-blind, Placebo-controlled study to evaluate the effect of *Lactobacillus* probiotic strain in the immune response in participants positive for SARS-CoV-2 infection	Probiotic *Lactobacillus salivarius* 1 × 10^9^ CFU + Vit D and + Zn—one capsule daily for 28 days	IgM and IgG anti-SARS CoV-2 antibodies + pro & anti inflammatory biomarker's measurement in 1 month
NCT04922918	Administration of *Ligilactobacillus salivarius* MP101 in an elderly nursing home during the COVID pandemic	Administration of *Ligilactobacillus salivarius* MP101 > 9 log 10 CFU daily for 4 months in fermented milk	Daily activities status and fecal and nasal immune profile 37 plex
NCT04907877	Role of nutritional support with Probiotics in Adults Outpatients with Symptomatic COVID-19: A randomized dietary study	NordBiotic ImmunoVir mixture: *Bifido* and *Lactobacteria* administered (5 billion) once a day for 28 days	Clinical symptom score of disease from healthy to worst outcome, hospitalization rate, time of recovery in 28 days' time frame, IgG response upto 6 months
NCT04884776	Modulation of Gut Microbiota to enhance Health and Immunity of vulnerable individuals during COVID-19 Pandemic	Probiotics-3 *Bifidobacteria* (10 billion CFU per sachet)-2 sachets/day for 12 weeks	IgG and IgM profile, change in gut microbiota level, inflammatory and glycaemic profile in 1, 6 and 12 months
NCT04877704	The effects of symprove, a multi-strain Probiotic, as an Adjuvant in the Management of COVID-19 in Hospitalized Patients	Symprove probiotics	Length of Hospital Stay, intestinal inflammation, symptoms recovery in 3 months
NCT04854941	Efficacy of probiotics (*Lactobacillus rhamnosus, Bifidobacterium bifidum, Bifidobacterium longum* subsp. *Infantis and Bifidobacterium longum*) in the treatment of hospitalized patients with Novel coronavirus infection	Probiotics with standard patient care & medication *Lactobacillus rhamnosus* PDV1705, *Bifidobacterium bifidum* PDV 0903, *Bifidobacterium longum* subsp. *infantis* PDV 1911 and *Bifidobacterium longum* PDV 2301 (10^9^ CFU each strain)-three times per day for 2 weeks	Disease prognosis, change in blood cells count, serum albumin, serum creatinine, CRP level, bilirubin, aminotransferase level and mortality during hospitalization
NCT04847349	Live Microbials to boost anti-SARSCoV-2 immunity clinical trial (Live basic trial)	Probiotic consortium-a capsule once a day for 21 days	Change in serum IgG, IgA and cytokine level in 21-42 days, reinfection upto 6 weeks

### Effects of antibiotics

All antibiotics including broad spectrum antibiotics like neomycin, vancomycin and metronidazole disrupt the microbiome function, thereby, activate the inflammasomes with signature of AP-1/nuclear receptor subfamily 4-group A (NR4A) to disrupt IgG 1 production (post influenza vaccine) and cause lung inflammation via M2 macrophages. The increased activation of Th1 cells and causing tissue pathology through chemokine receptor 1+ (CX3CR1+) in mononuclear phagocytosis (MNP)-dependent manner, which also alters the metabolome profile reducing the vaccine response [64]. The empirical antibiotics' treatments could lead to the loss of synbiotic bacteria in COVID-19 patients, supporting the fact of avoidance of unnecessary antibiotics use in the treatment of viral pneumonitis. Therefore, GM plays a significant role in antibiotics-host metabolism (xenobiotic) and disposition, which activates the prodrugs e.g., azo drugs to release sulfonamides [65].

The enzymes secreted by gut microbiota help metabolize the antibiotics to facilitate some important reactions e.g., acetylation, deacetylation, decarboxylation, dihydroxylation and demethylation to control the toxicity [65]. Given that the new dietary strategies could provide a clue to maintain homeostasis during COVID-19, to optimize the molecular mechanisms linked to health prospects.

### Vaccines

Vaccines induce variable response depending upon the population with different ages, nutritional status, immunological response and genetic disorders. Vaccines, nevertheless, regulate the GM to attenuate and strengthen the immune response fighting against various infections. The adjuvant properties of microbiota also increase the vaccine potency and vice-versa (Table 2). Microbiota on different vaccine administration sites may also play a significant role e.g., nucleotide binding oligomerization domain-containing protein 2 (NOD2) sensing is associated with intranasal route and IgA induction is mostly associated with attenuated influenza vaccine. The skin microflora influences the vaccines response given by intradermal route [10].

The live attenuated Bacillus Calmette Guerin (BCG) vaccine provides special protection against *Mycobacterium *spp. and is being used for many years reducing the infant mortality. The heterologous immune response exerted by this vaccine also protects against other respiratory infections [66]. This sort of protection develops in built memory through innate immune cells and reprogram the specific genes in monocytes through regulatory elements associated with histones directly linked to modulate the metabolic and epigenetic disorders, denoted as ‘trained immunity’. The monocyte cells (ex-vivo) were found to reprogram the promoter region of genes to produce anti-inflammatory cytokines to regulate the response through GM. Vaccine response may influence the adjuvant properties of GM that activate the innate and adaptive immune response [67]. The oral presence of *E. coli* in antibiotic treated GF mice had helped restoring the antibody response against influenza vaccine. The antibiotics driven dysbiosis may lead to dysregulate the immune response to vaccines, including BCG. In all cases, restoration of commensal microbiota reverted the impaired antibodies response [68].

BCG could be used to treat other ailments viz., bladder cancer, warts, leishmaniasis, candidiasis and asthma [66].

The serum treated GF mice have revealed that TLR5 were sourced from commensals microflora, responsible to enhance the immune response against influenza vaccine. Similar results obtained using polio and cholera vaccines, have shown a correlation between GM composition and development of  systemic and oral immunogenic response against vaccines in infants. The overall adjuvanting traits of commensals, nonetheless, are influenced by various factors such as, vaccine formulation, routes of immunization, to establish the immune regulation and GM stability [69]. Besides, the flagellin & peptidoglycan-muramyl dipeptide (MDP) acts agonist of NOD2 sensors to enhance its adjuvant properties against cholera toxin in mice. Similarly, the use of monophosphoryl lipid-A (MPL-A) of LPS to be recognized by TLR 4 could enhance the adaptive response during vaccination [69].

Lower abundance of *Bifidobacterium adolescentis* could reduce the NAb response using BNT162b2 vaccine. *Bifidobacteria* regulate the glycolysis pathways to produce lactate, acetate/or propionate being utilized by other commensals effectively, thus, maintain the gut balance to provide the high titre of NAbs against CoronaVac. Body mass index and body weight are responsible for reducing neutralization titre even with *Bifidobacterium adolescentis,  Butyricimonas virosa, Adlercreutzia equolifaciens* and *Asaccharobacter celatus* in CoronaVac vaccine [70]. *Ruminococcus torques, Eubacterium ventriosum* and *Streptococcus salivarius* are the high responders to vaccines. *Prevotella copri* and *Megamonas *spp. are associated with less adverse events. A higher prevalence of *Prevotella copri* was linked with farnesoid X receptor signaling via modulating bile acid metabolism [71]. *Megamonas funiformis* could ferment glucose into acetate and propionate to maintain homeostasis and *Megamonas hypermegale* could regulate the T17 helper cells [72]. *L. plantarum* is reported to increase the influenza specific IgA & IgG. The long-term implications of using these commensals to enhance vaccine efficacy and potency are yet to be revealed.

## Immunological dynamics

The discussion section is mainly based upon the immunological dynamics involving the immune cells and innate immune entities to modulate the response utilizing nutraceuticals and pharmacological agents through GM (Tables 1, 2 & 3, Figs. 1 & 2).

### Nutraceutical—immunity dynamics

The prospects of utilizing the probiotics as complementary therapeutics are being increased due to their adjuvant properties on dendritic cells DCs & NK-cells, and induce mucosal Abs secretion, regulate IFNs, produce SCFAs and MAMPs to engage the microbial sensors e.g., PPRs/TLRs regulating the CD4+ T-cells. Probiotics help improve intestinal metabolic and GM integrity and reduce the mitochondrial stress. Hence, mitigating the risks associated with respiratory infections with influenza, rhinovirus and respiratory syncytial virus [2, 27, 37]. Probiotic therapy has not yet been fully investigated and standardised in terms of safety and efficacy against COVID-19 and other infectious diseases.

The G-proteins coupled receptors (GPRs) react with SCFAs to activate the anti-inflammatory cascade, which inhibits IL-2 & upregulate IL-10 production in monocytes. It also controls the pro-inflammatory molecules like TNF-α, IL-1, but regulate NF-kB [28–30, 36].

There is an inverse correlation between high fibre diets and inflammatory markers of serum CRP, IL-6, IL-18, and TNF-α, thus attenuating the inflammasome complexes [31]. The SCFAs secreted through pre and probiotic treatments enter the immune cells and expressed in adipose tissues through GPRs-GPR 41 &43. The receptors GPR 41 and GPR 109 lead SCFAs to metabolize into acetyl CoA to regulate TCA cycle for cell function (Fig. 1) [32, 73–75]. SCFAs also inhibit the activity of histone deacetylase (HDAC) (Fig. 1) to control the process of deacetylation and histone crontonylation leading to acetylation involved in the post-translational modification, hence, regulating NF-kB [21, 28, 33, 36]. HDAC enzymes remove the acetyl groups from lysin residues in the NH2 terminal tails of core histones form more closed chromatin structure to repress the gene expression. Hence, SCFAs naturally help through nucleus to control HDACs facilitating the gene changes to regulate the chromatin architecture critical for epigenetic modification during cell cycle and differentiation. In this way, the immune cells keep themselves in active state during the antigen processing [32, 73, 74].

Treg cell differentiation also induced via SCFAs, as it plays a role as HADC-inhibitor to intensify the histone acetylation in Foxp3 and Il10 gene loci. It also impedes the stress of inflammatory macrophages and neutrophils being produced during infection. The aryl hydrocarbon receptors/ligands (AHR) were reported to develop innate lymphoid cells (ILC), especially, IL-22 and 3 ILC 3s [32]. The indole and IPA also modulate the cells to produce antibodies (Figs. 1 & 2).

Vitamin D via VDR receptors, induces the antimicrobial cathelicidin in macrophages and synthesize α-defensin to control viral replication by synthesizing antimicrobial peptides (AMPs) that damage the envelop structure of influenza A and respiratory syncytial viruses. It reduces the conc. of proinflammatory cytokines, but increase anti-inflammatory cytokines, therefore, increase apoptosis and autophagy through cellular and viral factors controlling the lymphocytopenia [38]. It maintains the intestinal integrity and eubiosis [38, 40, 42, 76]. Vitamin A, retinoic acid, also plays an important role in regulating the differentiation, maturation, and function of innate immune response.

The ω-3 PUFAs can be metabolized into a range of lipid mediators collectively called as ‘Specialized Pro-resolving mediators’ such as, prostaglandins, leukotrienes, thromboxanes, maresins, protectins and resolvins are utilized by the immune regulatory system [77]. The macrophage cells’ activities are influenced through ω-3 PUFAs, which add phospholipids in neutrophils. Dietary ω-3 PUFAs downregulate the IL-2 driven CD4+ and CD8+ T-cell activation and upregulate the Th2 CD4+ T-cells. Hence, the direct suppression of IL-2 induced Th1 cells, indirectly enhance the cross-regulatory function of Th2 cells exert anti-inflammatory effects (Fig. 1) [16].

Flavonoids could be useful during infection, as they increase Treg and T17 cell activity. They also suppress the TLR4, reduce the 1kB phosphorylation to reduce the activity of NF-kB [28, 36]. Flavonoids hinders the growth of pathogens-*Clostridium* spp., *Helicobacter pylori, Escherichia coli,* and *Salmonella typhimurium* [17].

### Pharmacological—immunity dynamics

The defeated IFN activities indicate the susceptibility to contract mycobacterial diseases. The interferon α-2 protein receptors i.e., IFNA2 and IFNA6 were reported to be upregulated in hospitalized patients. The IFNA2 is linked with IFN transcription in immune cells to produce cytokines and chemokines. Furthermore, IFNA2 activity decreases with seroconversion, but IFNA6 don’t (Fig. 1) [78]. SARSCoV-2 virus replication is also inhibited using IFNs in vitro (Vero and Calu3 cells), which induce the STAT1 phosphorylation in late infection [79].

Dexamethasone subsides the inflammatory cells and activates specific neutrophils against the virus, thereby, conforming the synergistic & potent therapeutic effects. Seropositive patients are not supposed to treat straightaway with antibody cocktail. Hence, the supplementation with dexamethasone was initiated in patients even who needed the oxygen support. Moreover, the anti-IL-6 (tocilizumab and sarilumab) is advised in patients who require oxygen having CRP level < 50 [80].

The antibiotics driven dysbiosis could alter the functions of many immune cells increasing the hyperactivity of intestinal macrophages and activation of pro-inflammatory helper T cells (Th) with depleted microflora, therefore, highly impeded SCFAs level increase the opportunity to contract more resistant infections [80]. The activation of M2 macrophages in lungs promote the allergic airway inflammation. The increased activation of Th1 cells causing tissue pathology through chemokine receptor 1 (CX3CR1+) & mononuclear phagocytosis (MNP)-dependent manner tend to alter the metabolome profile could diminish the vaccine response [64, 82]. GM metabolite IPA displays antibiotic properties against MDR *Mycobacterium *spp*.* and non-*Mycobacterial *spp*.*, which is a deamination analogue of tryptophan and acts as an allosteric inhibitor to the tryptophan secreted by *Mycobacterium* and blocks the synthesis of latter amino acids in vitro & in vivo [83].

The trained immunity produced by BCG could confer the protection against SARSCoV-2, including elderlies. It can also improve the protection against the HPV warts, increase the Ab production against influenza A (H1N1) and encephalomyocarditis, reduce the clinical symptoms with HSV virus to provide protection in experimental animals against HSV1&2 etc. [84]. The earlier studies have shown that BCG activates DCs & myeloid cells to increase I-III IFN and IL-28/29 production, and reduce the IFN-α and IFN-β production giving a strategy for establishing immunity against SARSCoV-2 [85]. The coronavirus associated gastroenteritis is controlled by *Enterococcus faecium* in piglets. The increased production of nitric oxide in the cells treated with *E. faecium* expressed IL-6 and IL-8 results in stimulating the cellular immunity to fight against TGEV infection [25].

TLRs/or PRRs and NOD-like receptors on DCs provide active protection via. adjuvanted GM. TLR-5 mediated signals yield effective Abs response against influenza vaccine [10]. Trimix, with CD70 and CD40 ligands and activated TLR4 (immune activator proteins) are recognized as effective adjuvant for mRNA to be encoded with similar sequences. The SCFAs induce higher Ab response via. effective stimulation of B-cells. Therefore, *Bifidobacterium adolescentis* was linked with higher NAbs titre in subjects who received CoronaVac to overcome the waning immunity of inactivated vaccines.

## Conclusion

It has been revealed that *L. rhamnosus* GG can prevent viral associated pneumonia (VAP) in high-risk ICU population [23]. More peer reviewed information is required to validate the efficacy of *L. rhamnosus* GG. Some studies, nonetheless, also suggest using probiotics could restrain other coronavirus associated gastroenteritis. The restoration of commensals may also revert the impaired antibodies response. The earlier studies have shown that BCG activates DCs & myeloid cells to increase IFN I-III and IL-28/29 production, and reduce the excessiveness of IFN-α and IFN-β giving a strategy for establishing immunity against SARSCoV-2 [85]. IFNs induce the STAT and JAK pathway to control viral infection in vitro. The open reading frames, ORF3b &ORF6, are antagonists to IFNs harbouring the diagnostic significance to inform disease prognosis, treatment options and animal model developments [79].

*Megamonas funiformis* and *Megamonas hypermegale* have been explored to sustain metabolic and T17 helper cells’ regulation [72]. Similarly, *L. plantarum* was reported to enhance influenza IgA and IgG.

The combinational therapy with pre and probiotics followed by vaccinations could have adjuvant effects in controlling the waning NAb titres. It is hypothesized that the future microbiota interventions may significantly improve the response to vaccines. *Bifidobacterium adolescentis* was linked with higher NAbs titre in subjects received CoronaVac to overcome the waning immunity of inactivated vaccines.

BNT162b2 vaccine elicited improved NAb response correlated to microbiota abundance with *Bifidobacterium and Roseburia *spp. [70]. Therefore, it is imperative the optimize involved therapies, while detecting microbial abundance. Probiotics and prebiotics supplements restore the diversity of gut microbiota, to regulate TLRs balancing the induction of pro-inflammatory and immune-regulatory cytokines conferring the viral clearance from lungs and gut to reduce the local and systemic pathogenicity. Hence, protect the body organs from permanent damage (Fig. 2) [20].

European food safety authority has already developed guidance in 2012 for safer use of *E. faecium* and implemented the viability assays revealing the complete protection against enteropathogenic coronavirus transmissible gastroenteritis virus (TGEV), in a dose-dependent rescue process [25]. Therefore, exploitation of these microbes offers the strategies to combat the viral gastroenteritis or IBD in humans.

Corticosteroids, vaccines, anti-sera, interferon therapies along with necessary nutraceutical supplements can enhance the protective immune response. Probiotic interventions at optimal dose level could minimize the debilitated effects in patients with improved symptomatic outcomes, therefore, shorten the stay in hospitals.

Some challenges are yet to be resolved e.g., over dose of probiotics can cause nausea and bloating. The comorbid status of patients should also be taken under consideration while using probiotics. The cohort study models should be designed to assess the errored immune signals, especially with people from underlying/comorbid conditions. Using risk free nutraceuticals trials irrespective of other medications’ regimens would certainly deliver various clues for personalized treatments to restore and sustain health. On the other hand, probiotics can also contribute to the antibiotic resistant genes, which may exchange thoroughly among commensals. This also provides clues to establishing strategies as how the health is impacted in clinic/hospital environment than at home. More research could be executed to study the impact of active foods on antibiotic susceptibility. A synergistic relationship among bacteriophages, essential probiotics, other commensals and how these implicate the immune cells modulation, including the environments contributing to the antibiotic resistance. Efficacies of ω-PUFA, other vitamins and dietary fibres along with probiotics is yet to be determined among different group of patients at different severity levels from asymptomatic to symptomatic. Last but not least, would probiotics really improve the potency of molecular vaccines to combat the problems of waning immunity, is yet to be revealed?

## Data Availability

Not applicable.

## Abbreviations

ARDS: Acute respiratory distress syndrome
AP1: Activator protein
AMP: Antimicrobial peptides
CCL3: Chemokine C–C motif ligand 3
CRP or hsCRP: C-reactive protein
Calu32B4 cells: Cellosaurus cell line
BCG: Bacillus Calmette Guerin
CCl2: Chemokine
CD40: Cluster of differentiation-40
DCs: Dendritic cells
DAT: Desaminotyrosine
DEX: Dexamethasone
DEFA5: Defensin α 5
FMF: Familial Mediterranean fever
GPXs: Glutathione peroxidases
GPR: G-protein coupled receptor
HDAC: Histone deacetylases
HSV: Herpes simplex virus
IL: Interleukin
IFNA: Interferon alpha protein
IFN: Interferon
LCHV: Bacteriocin acidocin
MAMPs: Microbe Associated Molecular Patterns
MCP-1: Monocyte chemoattractant protein-1
MIP-1α: Macrophage inflammatory protein-1α
MNP: Mononuclear phagocytosis
MMP7: Matrix Metalloproteinase 7
NK cells: Natural Killer cells
NF–kB: Nuclear factor kappa-light-chain-enhancer of activated B cells
NOD2: Nucleotide binding oligomerization domain-containing protein-1
NAb: Neutralizing antibodies
NR4A: Nuclear receptor subfamily 4, NOR1 group A related to neuron derived orphan receptor 1
ORF: Open reading frame
PRXs: Peroxiredoxins
PRR: Pattern Recognition Receptors
ROS: Reactive Oxygen Species activation
RdRp: RNA dependent RNA polymerase
SCFAs: Short chain fatty acids
SOD: Superoxide dismutase
STAT 1: Signal Transducer and Activation of Transcription
TNF-α: Tumour necrosis factor–α
Th cells: T helper cells
TGEV: Enteropathogenic coronavirus transmissible gastroenteritis virus
TjP: Tight junction protein
TCA: Tricarboxylic acid cycle
VAP: Viral associated pneumonia
VDR: Vit D receptors
ω-3 PUFAs: Omega-3-polyunsaturated fatty acids
